# Fungicidal activities of *Cymbopogon winterianus* against anthracnose of banana caused by *Colletotrichum musae*

**DOI:** 10.1038/s41598-023-33396-5

**Published:** 2023-04-24

**Authors:** Mark Anthony Angeles Mangoba, Dionisio de Guzman Alvindia

**Affiliations:** 1 Philippine Center for Postharvest Development and Mechanization, Department of Agriculture, Muñoz, Nueva Ecija Philippines; 2 Center for Natural Sciences and Environmental Research (CENSER), de La Salle University, Taft Ave., Manila, Philippines; 3 Department of Bio-Resource and Food Science, College of Life and Environmental Sciences, Konkuk University, Seoul, 143-701 South Korea

**Keywords:** Microbiology, Plant sciences

## Abstract

The genus Cymbopogon (Poaceae) species have been widely cultivated throughout the world for a wide range of uses in the pharmaceutical and agricultural fields. The current work investigates the fungicidal activities of *Cymbopogon winterianus* extract (CWE) in controlling the *C. musae* that caused anthracnose disease in banana fruit. In vitro assay results showed that CWE at 1.5–2.5 gL^−1^ concentrations controlled the development of the test pathogen. Mycelial blast, cytoplasmic discharge, and spore edema were noticed when CWE was applied. The Minimum Effective Concentration (MEC) of CWE for the in vivo assay was 1.50 gL^−1^ and can be used as a postharvest treatment on banana fruit to deter anthracnose infection. Moreover, no visible phytotoxicity or changes in aroma were observed on banana fruit treated with CWE, even at the highest concentration of 2.5 gL^−1^. The GCMS analysis revealed 41 chemical components associated with CWE. The five main compounds were the following: Methyl oleyl ether (40.20%), γ-Sitosterol (15.80%), 6-Methylheptan-3-ol (7.13%), α-Terpineol (5.56%), and n-Pentadecanol (4.05%). The CWE possesses excellent fungicidal effects against *C. musae*; in the near future, it can be used as an alternative to commercially available traditional fungicides on the market.

## Introduction

Philippine banana farming practices have significantly transitioned due to consumer preference for sustainable agricultural products. As an illustration, most of the banana farms in the Philippines have converted land to produce “organic” bananas. Similarly, small-scale farmers cultivate bananas in their backyards without using synthetic fungicides during the growing process or after harvest. Plantation-grown organic and non-chemical bananas are typically exported to various Asian nations. Furthermore, most consumers choose organic bananas because they are free of agricultural synthetic chemicals.

Bananas have been arriving on the market with substandard quality due to postharvest disease, mainly anthracnose, owing to the lack of synthetic fungicide treatments available^1^. *Colletotrichum* species can cause anthracnose, but the most prevalent species is *Colletotrichum musae*, which results in 30–40% losses in commercial value^2,3^. This fungus infects young banana fruit but goes unrecognized until the fruit turns ripe and yellow^4^.

On the other hand, earlier studies have shown that natural products (herbs, spices, ornamentals, and essential oil) and other edible coatings (chitosan) are effective against pests and diseases of agricultural commodities^5–16^. Various *Cymbopogon* species have been widely cultivated throughout the world for a wide range of uses, which include antifungal^17^, insecticidal^18^, antibacterial^19^, and pharmaceutical^20^. However, little evidence is available regarding the efficacy of *Cymbopogon winterianus* extract (CWE) in controlling the *C. musae* that causes anthracnose disease in bananas. Further, the availability of raw plant materials of *C. winterianus* in the trading area in tropical and subtropical regions of Asia, America, and Africa was the basis for choosing it as a test plant. Also, the current investigation was to establish the fungicidal impact of CWE in managing anthracnose diseases in banana fruit*.* The information gathered could be used to treat anthracnose infection in banana fruit and a potential replacement for synthetic fungicides sold commercially soon.

## Results

**Figure 1:**
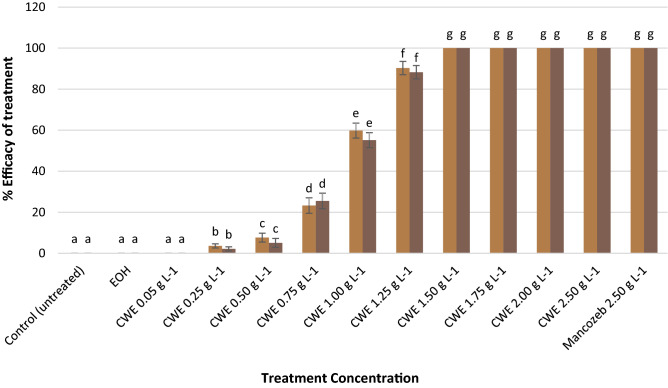
CWE's effect on *C. musae* mycelial development seven days after treatment. Trials 1 and 2 are illustrated by the dark and light bars, respectively. Based on the Tukey test, values followed by similar letters are not statistically different.

**Figure 2:**
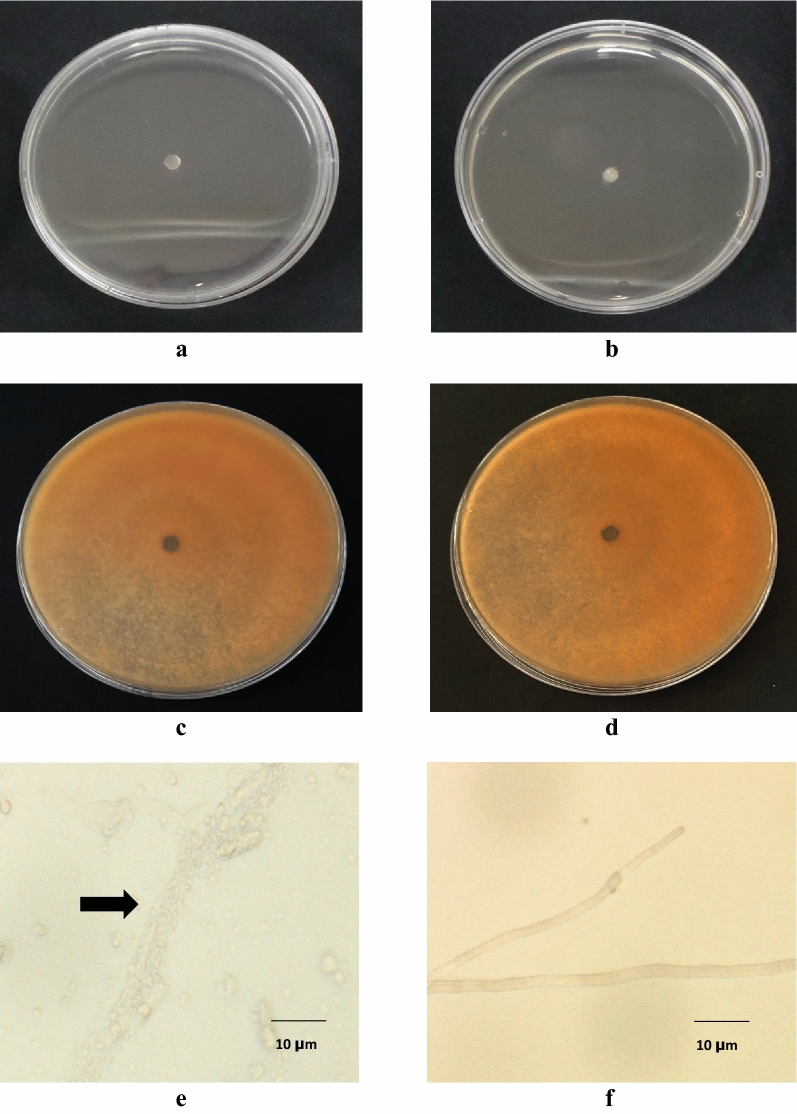
Test pathogen treated with (**a**) CWE at 1.50 gL^−1^, (**b**) Mancozeb 2.50 gL^−1^, (**c**) EOH, (**d**) untreated mycelial plug, (**e**) mycelia treated with CWE, (**f**) untreated mycelia.

The effects of CWE on *C. musae* mycelial growth (in-vitro) were summarized in Fig. 1. The CWE completely suppressed the mycelial growth of *C. musae* at a minimum rate of 1.50 gL^−1^ (Fig. 2a), which was statistically equal to Mancozeb's 2.50 gL^−1^ (Fig. 2b). Mycelial plug treated with EOH and the untreated control showed no significant effect on the C. musae (Fig. 2c and d). On the other hand, the fungal pathogen's interaction with CWE resulted in a mycelial burst (Fig. 2e). However, the untreated *C. musae* showed more robust mycelial development (Fig. 2f).

**Figure 3:**
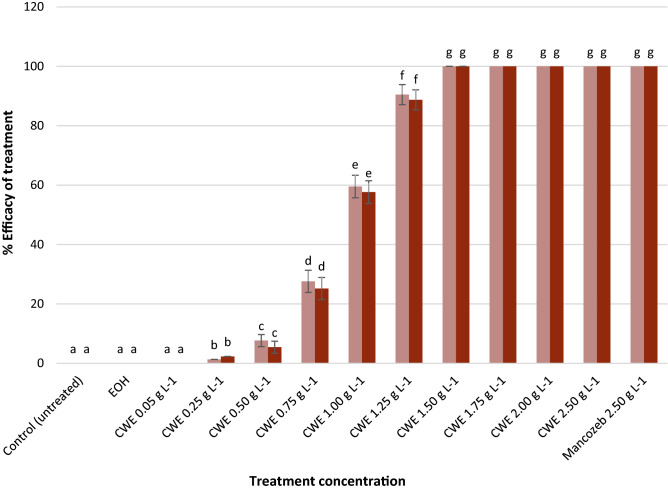
The efficacy of CWE on spore germination of *C. musae.* Trials 1 and 2 are shown by the light and dark bars, respectively. Based on the Tukey test, values followed by similar letters are not statistically different.

**Figure 4:**
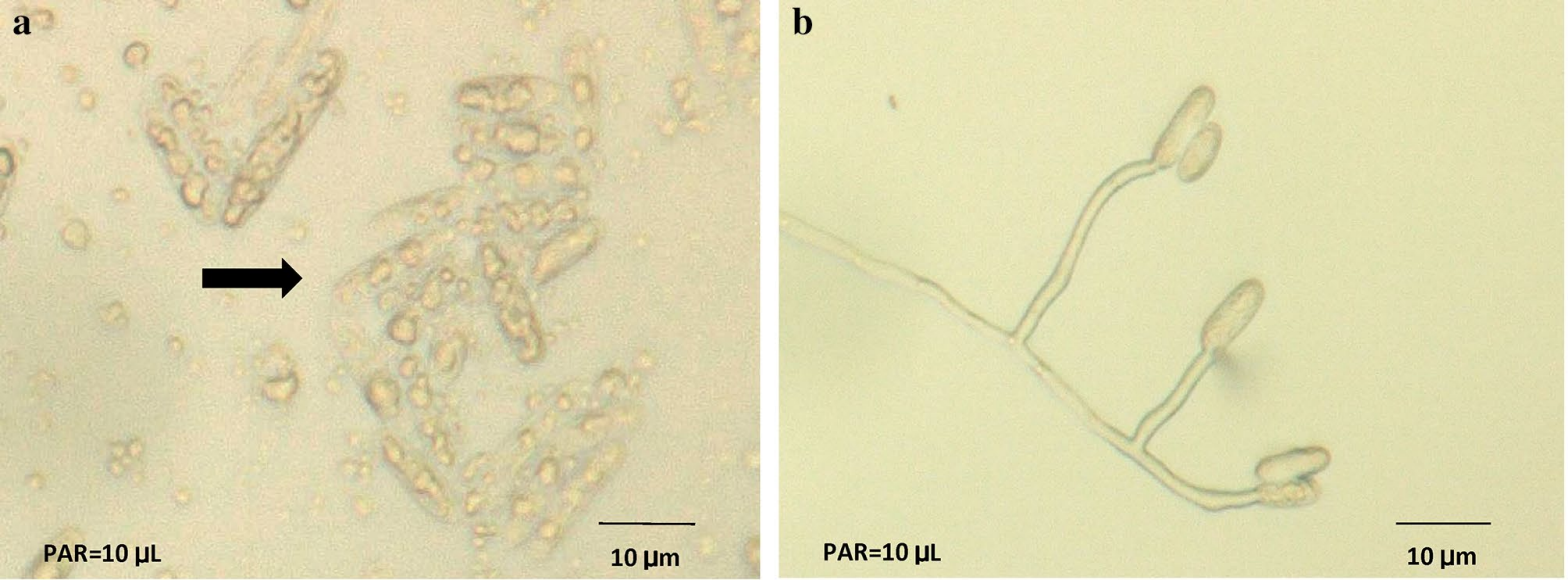
Spore of *C. musae* (**a**) treated with CWE, (**b**) untreated, 48 h after treatment application.

The CWE significantly inhibited the spore germination of *C. musae* at a rate of 1.50 gL^−1^ (Fig. 3). Further, spore edema and cytoplasmic discharge (as pointed in the arrow) were noticed in *C. musae* treated with CWE (Fig. 4a), but germinated spores were observed in the untreated control (Fig. 4b).

**Figure 5:**
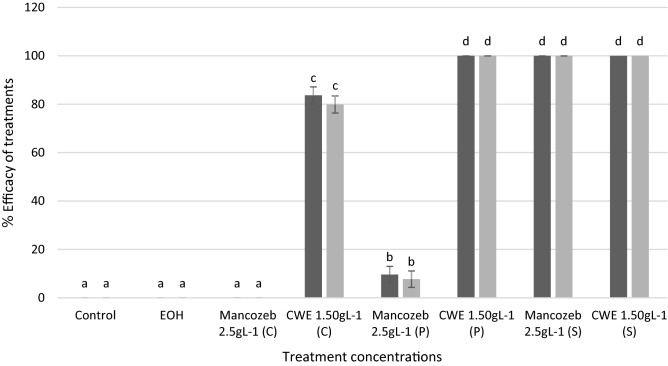
The response of *C. musae* to various treatments (in-vivo). Trials 1 and 2 are shown by the red and orange bars, respectively. According to the Tukey test results, values followed by similar letters are not statistically different from one another. (C) = curative, (P) = preventative, (S) = simultaneous tests.

The application of CWE 24 h before, after, or simultaneously administered with the fungal pathogen (in-vivo) was summarized in Fig. 5. The result showed that CWE was much more effective than synthetic fungicides in suppressing anthracnose disease after seven days. The curative treatment of the CWE at 1.50 gL^−1^ (76.93–80.86%) was considerably better than Mancozeb at 2.50 gL^−1^, which did not affect the growth of *C. musae* on the banana fruit surface. However, the preventative effects of CWE at a concentration of 1.50 gL^−1^ demonstrated complete control against anthracnose infection, while the efficacy given by Mancozeb was only 7.72–9.62%. On the other hand, CWE was statistically equal to a synthetic fungicide with a 100% inhibitory effect on a fungal pathogen in a simultaneous assay.

**Figure 6:**
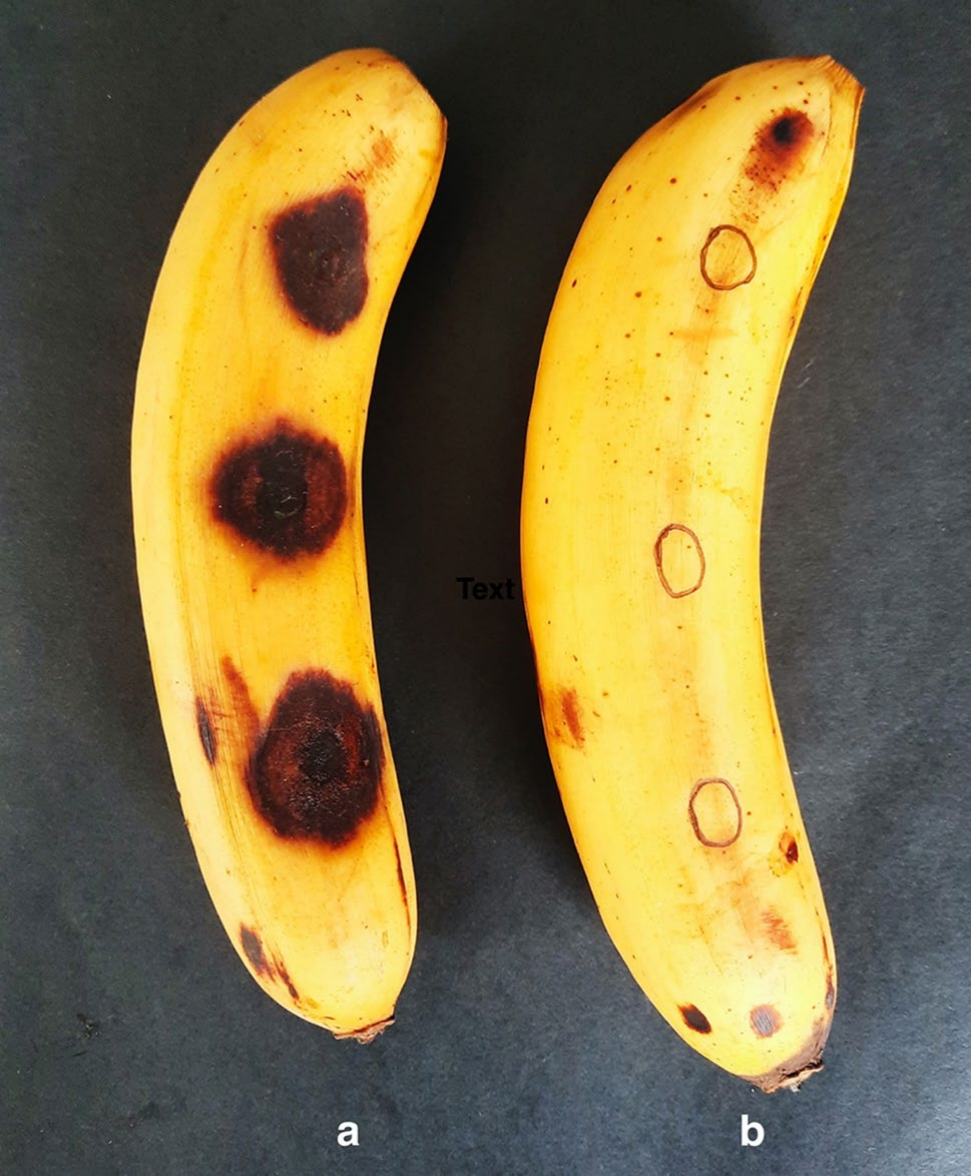
Banana fruit treated with (**a**) *C. musae* suspension and (**b**) CWE, after seven days.

Meanwhile, banana fruit inoculated with 10^8^ mL^−1^ *C. musae* suspension developed black lesions on the fruit's surface (Fig. 6a). However, even at the highest concentration of 2.5 gL^−1^ CWE, treated banana fruit showed no discoloration and no detectable phytotoxicity after 7 d (Fig. 6b).

The GCMS analysis revealed forty-one chemical components associated with CWE (Table 1). The following were the five main compounds of CWE: Methyl oleyl ether (40.20%), γ-Sitosterol (15.80%), 6-Methylheptan-3-ol (7.13%), α-Terpineol (5.56%), and n-Pentadecanol (4.05%).

**Table 1:** Chemical compositions of CWE.

RT	NAME	% Area
4.859	2.6-Dimethyl-6-nitro-2-hepten-4-one	0.41
5.082	2,2-Dimethyl-3-pentanol	0.15
5.176	6-Methylheptan-3-ol	7.13
5.356	2.4.4.6-Tetramethyl-(1.3.2)dioxaborinane	0.36
5.773	2.6-Heptanedione 3-acetyl	0.33
5.817	Linalool, Oxide	0.12
5.864	Linalool, Oxide	0.14
7.994	α-Terpineol	5.56
11.36	Tetradecanoic acid	0.42
13.673	n-Pentadecanol	4.05
15.2	n-Hexadecanoic acid	2.11
17.11	9.12-Octadecadien-1-ol.(Z.Z)	1.27
17.272	Oleyl alcohol,methyl ether	40.20
17.382	Oleyl alcohol	1.76
17.77	1-Octadecanol methyl ether	1.10
19.608	Hexadecanamide	0.41
21.386	Tridecanedial	0.70
26.081	Hexadecanoic acid 2-hydroxy-1-(hydroxymethyl)ethyl ester	1.63
28.751	Octadecanoic acid 2 3-dihydroxypropyl ester	0.92
29.358	Erucamide	0.44
30.541	Heptacosyl heptafluorobutyrate	0.33
32.378	Cholesta-4 6-dien-3-ol (3.beta.)-	1.46
32.775	1-Hexacosanol	2.28
32.983	Cholesterol	1.35
34.502	Campesterol	2.18
35.982	γ-Sitosterol	15.80
36.666	9,19-Cyclo-9.beta.-lanostane-3.beta.25-diol	1.83
37.065	4.4.6a.6b.8a.11.12.14b-Octamethyl-1.4.4a.5.6.6.6a.6b.7.8.8a.9.10.11.1	2.15
37.691	24-Norursa-3.12-diene	3.41

## Discussions

In general, the CWE successfully suppressed the development of *C. musae*. Furthermore, it substantially reduced and even controlled the anthracnose development in banana fruit when administered with CWE as a postharvest treatment. The previous studies on using natural products against postharvest diseases observed similar results^21–25^. The CWE had a minimum effective concentration (MEC) of 1.50 gL^−1^ in managing anthracnose infection, and it was observed at this dose with no significant phytotoxicity on banana fruit. On the other hand, the *C. winterianus* is well-known for its strong scent, primarily due to the presence of monoterpene compounds. However, under the phytotoxicity test results, the scent of CWE is only recognizable in the first 48 h after application; thereafter, no CWE aroma was detected. Thus, the quality can be acceptable and recommendable to consumers' preferences. Moreover, these monoterpene compounds were naturally degraded by the environment^26^.

The CWE, on the other hand, showed dose dependence, with the higher concentration having a more significant effect. According to the present data, the volume content of CWE at 1.50 gL^−1^ was 1.67 times lower than the volume content of the synthetic chemical fungicide (Mancozeb at 2.5 gL^−1^) which is necessary to achieve control. The main components and minor derivatives of CWE worked together to make it even more toxic, causing the target species to die quickly.

The variations in treatment responses between in vivo and in vitro were explained by the absorption capacity and degree of complexity of two distinct surfaces: agar (in vitro) and banana fruit (in vivo). In an in-vivo test, the fruit's physiological properties cooperate with each other, causing treatments with moderate to high toxicity against *C. musae*^27–29^. Based on the results of a preventative assay, *C. musae* may possibly have developed Mancozeb resistance. Other studies have shown that major fungal pathogens of fruit are also resistant to synthetic chemical fungicides such as Mancozeb^30^. Meanwhile, the findings of the preventative test outperformed those of the curative assay in terms of treatment efficacy. This could be due to the fact that *C. musae* had colonized the surface of the banana fruit prior to treatment application.

Two of CWE's five key components have shown antimicrobial properties: γ-sitosterol and α-Terpineol^31,32^. So far, no biological activities on microorganisms have been reported for oleyl alcohol.methyl ether, 6-Methylheptan-3-ol, and n-Pentadecanol,

Meanwhile, there are two significant benefits associated with using plant extracts as fungicides. For example, most extracts derived from plants do not pose health risks, and in most cases, they are beneficial to the environment^33–36^. Moreover, there is a decrease in the development of chemical resistance. In some cases, a microorganism can develop resistance to a single compound. However, it is much more difficult for microorganisms to develop resistance to multiple or complex compounds^36–38^. Because various microorganisms have different responses to the same compound, and multiple compounds have a significant potential to increase the toxicity they have on the target microorganisms they are trying to kill^39^. In addition to this, C. *winterianus* is readily available on the market. On the other hand, solvent and the equipment needed for extraction are also commercially available, and processing does not present any tough challenges. As a result, small-to-medium-scale banana farmers may be able to use it to control anthracnose infection in banana fruit.

Meanwhile, future research should focus on the interactive effect of CWE in combination with other natural products. The CWE storage studies may also be required. Other aspects of these natural products, such as the mechanism of action and formulation, should be addressed to ensure optimal stability and potency.

## Methods

### Preparation and chemical profiling of CWE

The *C. winterianus* samples were collected in Nueva Ecija, Philippines 15° 35′ N  121° 00′ E. The aerial portion of CWE was extracted using Mangoba and Alvindia's methodology^9–11^. The CWE was evaporated using a USA Lab RE-1020 rotary evaporator. The extracts were immediately stored in an ultralow freezer at − 80 °C until biological assays and chemical analyses were performed. On the other hand, the chemical profiling of CWE was carried out according to the Alvindia and Mangoba^11^ protocol using a Shimadzu GC–MS. Furthermore, Shimadzu Gas Chromatography (GC) and Mass Spectrometer (MS) models GC2010 and QP2010 were used. Furthermore, the individual components of the CWE were characterized using the National Institute of Standards and Technology's system library's fragmentation display of mass spectra and retention time (RT). The peak area of the GC was used to calculate the percentage of each compound in the CWE.

### The test pathogen

The *C. musae* culture was acquired from the laboratory of Dionysus Enterprises, located in Nueva Ecija, Philippines. The test pathogen was previously isolated from naturally infected Cavendish banana fruit, which exhibits anthracnose lesions^9–11^. The pure isolated culture of *C. musae* was kept at 6 °C on potato dextrose agar (PDA). The pathogenicity of *C. musae* was maintained and tested on banana fruit by means of artificial inoculation and periodic re-isolation.

### In-vitro assay

The efficacy of CWE on the mycelial growth of *C. musae* was conducted using the method described by Alvindia and Mangoba^9,10^. PDA was homogenized with CWE at dosages ranging from 0.05 to 2.50 gL^−1^. The PDA plates that had not been treated served as a negative control, whereas the PDA plates that had been treated with a commercial fungicide (Mancozeb at 2.5 gL^−1^) served as a positive control. Further, the *C. musae* mycelial plug (5 mm) was put into the center of treated PDA plates for seven days and stored at 25 ± 2 ℃ and 70 ± 5% relative humidity (RH). The growth of *C. musae* was assessed after seven days by measuring the colony diameter using a digital caliper. The test was done twice, with three replications per treatment, and was set up in a completely randomized design (CRD). The percent efficacy of treatment (%ET) was computed as follows: (CNC-CDT)/CNC X 100, where CNC is the negative control colony diameter, and CDT is the treated plug colony diameter.

The spore germination assay was conducted following the Alvindia and Mangoba procedures^9–11^. CWE (0.05–2.50 gL^−1^) was applied to a 10 μL fungal suspension containing 10^8^ spores mL^−1^ and evenly dispensed on water agar. Under the compound microscope, 100 spores were counted after 48 h. The percent efficacy of treatment (% ET) for spore germination was calculated using the method recently described by Alvindia and Mangoba^9–11^. Spore dilatation and burst were noted throughout the study. The experiment was repeated twice with a tri-replicate for each treatment and was arranged as mentioned earlier.

### In vivo assay

Cavendish banana samples were collected in Davao del Norte, Philippines 7°21′N 125°42′E. The owner of the banana plantation, Mr. Eduardo Madrid, gave permission to take samples during the study. The fruit (80% maturity) was de-handed and rinsed in sterile water before removing the crown. The fruit was taken from a single banana tree to ensure the homogeneity of the test material. Each treatment is comprised of six banana fingers. The banana finger has three inoculation sites: tip, center, and bottom. Each inoculation site was marked and partially injured with an insect needle (No. 1). The insect needle was stabbed once into the banana fruit skin, leaving a ≦1.50 mm diameter wound. Following that, 10μL of a spore solution of *C. musae* [10d old (10^8^ spores mL^−1^)] and treatments (CWE and Mancozeb) were administered to each inoculating site either 24 h before (preventative test), simultaneously applied or after 24 h (curative test). Control fruit was solely applied with *C. musae* spore suspension. The treated banana fingers were maintained in a sterile plastic box (25 X 40 cm) at 25 ± 2 ℃ and 70 ± 5% RH. After seven days, the treatment impact on *C. musae* was evaluated using a digital caliper to measure the lesion diameter on the fruit surface. The treatments' percent effectiveness was calculated as previously stated. Further, the phytotoxicity of CWE and the pathogenicity of *C. musae* on banana fruit were recorded. The experiment was done twice with six replicates per treatment and arranged using CRD.

### Statistical analysis

An analysis of variance (ANOVA) was undertaken on all data using SPSS Statistics version 16.0 for IOS. Significant differences between treatments were determined using the Tukey HSD test (IBM 20 for IOS, NY, USA).

## Data Availability

The datasets generated and/or analyzed during the conduct of the study are not publicly available due to internal policy and protocol but are available from the corresponding author on reasonable request.
